# Multiparameter mapping of relaxation (R1, R2*), proton density and magnetization transfer saturation at 3 T: A multicenter dual‐vendor reproducibility and repeatability study

**DOI:** 10.1002/hbm.25122

**Published:** 2020-07-08

**Authors:** Tobias Leutritz, Maryam Seif, Gunther Helms, Rebecca S Samson, Armin Curt, Patrick Freund, Nikolaus Weiskopf

**Affiliations:** ^1^ Department of Neurophysics Max Planck Institute for Human Cognitive and Brain Sciences Leipzig Germany; ^2^ Spinal Cord Injury Center University Hospital Balgrist, University of Zurich Zurich Switzerland; ^3^ Medical Radiation Physics, Department of Clinical Sciences Lund Lund University Lund Sweden; ^4^ NMR Research Unit, Queen Square MS Centre, Department of Neuroinflammation UCL Queen Square Institute of Neurology, University College London London UK; ^5^ Wellcome Centre for Human Neuroimaging UCL Queen Square Institute of Neurology, University College London London UK; ^6^ Department of Brain Repair & Rehabilitation UCL Queen Square Institute of Neurology, University College London London UK; ^7^ Felix Bloch Institute for Solid State Physics, Faculty of Physics and Earth Sciences Leipzig University Leipzig Germany

**Keywords:** clinical trial, in vivo histology using MRI, multicenter study, multiparameter mapping, quantitative MRI, reproducibility

## Abstract

Multicenter clinical and quantitative magnetic resonance imaging (qMRI) studies require a high degree of reproducibility across different sites and scanner manufacturers, as well as time points. We therefore implemented a multiparameter mapping (MPM) protocol based on vendor's product sequences and demonstrate its repeatability and reproducibility for whole‐brain coverage. Within ~20 min, four MPM metrics (magnetization transfer saturation [MT], proton density [PD], longitudinal [R1], and effective transverse [R2*] relaxation rates) were measured using an optimized 1 mm isotropic resolution protocol on six 3 T MRI scanners from two different vendors. The same five healthy participants underwent two scanning sessions, on the same scanner, at each site. MPM metrics were calculated using the hMRI‐toolbox. To account for different MT pulses used by each vendor, we linearly scaled the MT values to harmonize them across vendors. To determine longitudinal repeatability and inter‐site comparability, the intra‐site (i.e., scan‐rescan experiment) coefficient of variation (CoV), inter‐site CoV, and bias across sites were estimated. For MT, R1, and PD, the intra‐ and inter‐site CoV was between 4 and 10% across sites and scan time points for intracranial gray and white matter. A higher intra‐site CoV (16%) was observed in R2* maps. The inter‐site bias was below 5% for all parameters. In conclusion, the MPM protocol yielded reliable quantitative maps at high resolution with a short acquisition time. The high reproducibility of MPM metrics across sites and scan time points combined with its tissue microstructure sensitivity facilitates longitudinal multicenter imaging studies targeting microstructural changes, for example, as a quantitative MRI biomarker for interventional clinical trials.

## Graphical abstract

We developed and optimized a quantitative acquisition technique, which enables the fast high‐resolution mapping of multiple parameters on standard clinical MRI hard‐ and software. A travelling heads study on five healthy volunteers demonstrated its excellent reproducibility and comparability across six different sites with MRI scanners from two different vendors. The low inter‐site bias (<5%) for multicenter trials and the low intra‐scan variances (between 4 and 10% for R1, PD, and MT) suits it ideally for multi‐center longitudinal studies—such as clinical interventional studies.

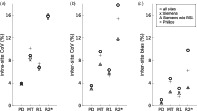

## 1 INTRODUCTION

Multicenter clinical studies using magnetic resonance imaging (MRI) increase the size and diversity of the study population (Van Horn & Toga, 2009). Quantitative MRI (qMRI) can improve comparability across time points and sites (Cercignani, Dowell, & Tofts, 2018; Weiskopf et al., 2013). Moreover, qMRI metrics allow the tissue microstructure, such as myelin, iron, and the neuropil, to be indirectly probed, by separating disparate contrast mechanisms unlike standard contrast‐weighted MRI (Edwards, Kirilina, Mohammadi, & Weiskopf, 2018; Weiskopf, Mohammadi, Lutti, & Callaghan, 2015). Thus, qMRI has been applied in several multicenter studies (Weiskopf et al., 2013) and clinical trials, providing imaging biomarkers in the development of new outcome measures (Deoni et al., 2008; Filippi & Agosta, 2007; Freund et al., 2019; Horsfield et al., 2003; Mahajan & Ontaneda, 2017; Willcocks et al., 2016).

The multiparameter mapping (MPM) method (Helms, Dathe, Kallenberg, & Dechent, 2008; Weiskopf et al., 2013) provides quantitative high‐resolution maps (magnetization transfer saturation [MT], proton density [PD], longitudinal and effective transverse relaxation [R1, R2*]) sensitive to myelin and iron content in the brain within clinically feasible scan times (~20 min). MPM has been applied in a wide range of studies to investigate microstructural tissue properties in gray and white matter and has proven its potential to provide quantitative imaging biomarkers (Callaghan et al., 2014; Freund et al., 2013; Grabher et al., 2015; Seif et al., 2018; Weiskopf et al., 2013; Ziegler et al., 2018).

A previous multicenter study applying the MPM technique showed reproducibility of the quantitative maps using custom‐made sequences on the same MRI scanner model (Siemens Trio, software version VB17) (Weiskopf et al., 2013). Moreover, MPM has been applied in longitudinal studies to investigate microstructural brain changes induced by spinal cord injury (SCI) (Freund et al., 2013; Grabher et al., 2015; Villiger et al., 2015). Based on the reported inter‐site comparability and sensitivity to brain changes, the MPM protocol is currently being applied as an MRI outcome measure in an SCI clinical trial. More specifically, the multicenter, multinational, placebo‐controlled, phase‐II clinical trial NISCI (www.nisci-2020.eu) is using MPM to investigate the safety and preliminary efficacy of intrathecal anti‐Nogo‐A [NG101] in patients with acute SCI (Kucher et al., 2018). However, implementing a qMRI protocol in multicenter studies such as the NISCI trial requires careful coordination. Considerations involve how differences in scanner hardware and software can influence the MRI outcome measures and potentially lead to conflicting results.

This study therefore aimed (a) to optimize the MPM protocol and processing pipeline based on the vendors' product sequences customized for clinical trials (rather than custom sequences) and (b) to test the protocol across different MRI scanner types in the form of a traveling heads study. In this article we report the scan‐rescan repeatability and inter‐site comparability of the MPM protocol across six different clinical sites involved in the NISCI trial.

## 2 METHODS

### 2.1 Subjects and sites

The study was conducted on six 3 T MRI systems with different hardware and software (Table 1). Four scanners were manufactured by Siemens Healthineers (Erlangen, Germany) and two by Philips Healthcare (Eindhoven, Netherlands). Five healthy subjects (2 female, 3 male, aged 32.4 ± 6.0 years [mean ± *SD*]) were scanned twice each (i.e., scan‐rescan) at each site with an average inter‐scan interval of 2 hr between measurements. Informed written consent was obtained from each subject prior to each scan, and all sites obtained local ethical approval. Local radiographers, who were also involved in the NISCI study, where possible, performed the scans.

**TABLE 1:** MRI scanning sites and main hardware and software specifications

Site	MRI vendor	MRI system	Number of channels of RF receive head coil	MRI software version
BCN	Siemens	Verio	32	VD13A
BSL	Siemens	Prisma	20	VE11C
HD	Siemens	Verio	16	VB19A
NOT	Philips	Achieva	16	5.3.0
ZH	Siemens	Skyra	16	VE11B
SNS	Philips	Achieva	16	5.1.7

*Note:* BCN: Clinica Creu Blanca, Barcelona, Spain; BSL: Radiology Department, University Hospital Basel, Basel, Switzerland; HD: Spinal Cord Injury Center, Univerisity Hospital, Heidelberg, Germany; NOT: Radiology, Swiss Paraplegic Center, Nottwill, Switzerland; ZH: Spinal Cord Injury Center, University Hospital Balgrist, University of Zurich, Zurich, Switzerland; SNS: Laboratory for Social and Neural Systems Research, Zurich Center for Neuroeconomics, University of Zurich, Zurich, Switzerland.

### 2.2 MRI acquisition

The MPM protocol was implemented based on product sequences available on the manufacturer's clinical MRI systems. Three‐dimensional (3D) data acquisition was composed of three multi‐echo spoiled gradient echo scans (i.e., fast low angle shot [FLASH] sequences on Siemens scanners and multi‐echo fast field echo [mFFE] sequences on Philips scanners) with MT, T1, and PD contrast weightings. Additional reference scans for bias correction using the hMRI‐toolbox (RRID: SCR_007037) (Tabelow et al., 2019) included mapping of the radio‐frequency (RF) transmit (B1^+^) and receive fields (B1^−^) on both vendor scanner platforms. The total acquisition time was 18:45 min on the Siemens scanners and 23:58 min on the Philips scanners.

Generally, the parameters of the multi‐echo gradient echo sequences were chosen with the aim of keeping scan time short (~20 min) and to achieve an ~1 mm isotropic resolution with a high image quality. A protocol previously used in a study on spinal cord injury served as the starting point for the protocol optimization, since it allowed successful monitoring of longitudinal injury related changes (Freund et al., 2013). The total number of acquired echoes, repetition time (TR), and maximum echo time (TE) were reduced to further shorten the scan time, while still allowing for reliable mapping of R2* in subcortical areas with typically high R2* values. The spacing between the different echoes was determined by using a high readout bandwidth of 480 Hz/pixel, in order to minimize chemical shift artifacts and achieve a high number of echoes for improved signal decay modeling. The excitation flip angles of the T1‐ and PD‐weighted gradient echo sequences were based on the median nominal Ernst angle for brain tissue, scaled by a factor of 0.4142 and 2.4142, respectively, in order to minimize noise propagation into the maps of the brain (Dathe & Helms, 2010). In the MT‐weighted gradient echo sequence, a small constant flip angle of 6° was chosen to control its systematic influence on the MT maps (Helms, Dathe, Weiskopf, & Dechent, 2011). The parameters of the MT saturation pulse were limited to the vendors' default product sequence settings (Table 2), since changes would have required pulse sequence programming and would not have been feasible for a clinical study. MT values were harmonized across vendor platforms in the postprocessing. The minimum TR for MT‐weighted sequences on the Philips platforms was driven by specific absorption rate (SAR) constraints. The RF spoiling characteristics differ between vendors' sequence implementations (Table 2), which was accounted for in the postprocessing.

**TABLE 2:** Different MRI parameters on the Philips and Siemens scanners

*Acquisition parameter*	*Siemens*	*Philips*
*Minimum TE and ΔTE* (ms)	*2.46*	*2.40*
*Maximum TE* (ms)	*14.76*	*14.40*
*Acquisition resolution* (mm^3^)	*1.1 × 1.0 × 1.1*	*1.0 × 1.0 × 1.0*
*FoV slice resolution* (%)	*91.0*	*78.5*
*FoV phase* (%)	*87.50*	*93.75*
*Slice pF* (%)	*75.0*	*62.5*
*Phase R*	*2*	*2*
*Slice R*	*1.00*	*1.25*
*PD & T1 TA* (min)	*3:38*	*3:37*
*Spoiling phase increment* (°)	50*	150*
*MT TA* (min)	*6:37*	*9:40*
*MT TR* (ms)	*37*	*48*
*MT pulse angle* (°)	500*	220*
*MT pulse length* (ms)	10*	8*
*MT pulse off res. freq*. (kHz)	1.2*	1.0*
*MT pulse shape*	Gaussian*	Gauss filtered sinc*
*MT pulse bandwidth* (Hz)	192*	300*

*Note:* Default settings (mostly unchangeable) marked with *.

Abbreviations: FoV, field of view; MT, magnetization transfer; PD, proton density; pF, partial Fourier; R, parallel speedup factor; T1, longitudinal relaxation; TA: acquisition time; TE, echo time; TR, repetition time.

The acquisition protocols shared the following common parameters across all platforms (see Table 2 for differing parameters): TR of PD‐ and T1‐weighted contrasts: 18 ms; flip angles for MT, PD, and T1 weighted contrasts: 6°, 4°, 25°, respectively; six equidistant echoes (for TE, see Table 2); 1 mm isotropic reconstruction voxel size; readout (RO) field of view (FoV): 256 mm; base resolution: 256 pixels in the RO direction; 176 slices; readout in the head‐foot direction, inner phase encoding loop in the left–right (“slice”) direction, outer phase encoding loop in the anterior–posterior direction (“phase”); RO bandwidth: 480 Hz/pixel; elliptical k‐space coverage; parallel imaging speedup factor of 2 in the slow phase encoding direction (comprehensive list of parameters in Supplement 1).

The B1^+^ field mapping methods differed across vendors and sites. At Siemens sites, vendor‐supplied sequences were used. At three sites (BCN, HD, ZH) a rather slow (2:14 min) implementation “rf map” was applied. It was based on spin‐echo and stimulated echo acquisitions and is similar to the customized sequence by Lutti, Hutton, Finsterbusch, Helms, and Weiskopf (2010). However, it used a 2D gradient echo readout instead of a 3D echo‐planar imaging (EPI) readout. At another Siemens site (BSL), we used a faster implementation (“tfl_b1map”) (12 s) utilizing a gradient echo sequence with ultrafast turbo‐FLASH readout (available from version VE11 onwards) (Chung, Kim, Breton, & Axel, 2010). At the Philips sites (NOT, SNS) a vendor implementation of the actual flip angle imaging (AFI) technique was used (Yarnykh, 2007, 2010), which acquires spoiled gradient echo signals with two alternating TRs (3:36 min total scan time). The B1^+^ mapping acquisition parameters are detailed in Supplement 1.

The high‐resolution data was corrected for apparent sensitivity changes due to head motion between the acquisitions of the three differently weighted volumes, as implemented in the hMRI‐toolbox (Papp, Callaghan, Meyer, Buckley, & Weiskopf, 2016; Tabelow et al., 2019). To this end, low‐resolution 3D spoiled gradient echo volumes were acquired twice: once with the RF head coil and once with the body coil, with acquisition times of 10 s per coil. The ratio provided a relative net RF receive field sensitivity (B1^−^) map of the head coil (Papp et al., 2016; Tabelow et al., 2019). The acquisition was optimized for speed by using a low isotropic spatial resolution of 4 mm, short TE (2–3 ms) and a low flip angle of 6° (no partial Fourier, no parallel imaging speedup). The acquisition of the head and body coil volume pair was repeated before each of the three MPM contrasts (MT, PD, and T1). On the Philips platform, the sensitivity estimate and correction was performed in addition to the pre‐scan procedure (multi‐channel RF coil sensitivity normalization; “CLEAR”), since the built‐in procedure typically acquires the sensitivity maps once and does not dynamically update them between scans (Papp et al., 2016).

### 2.3 Data quality control

The acquisition parameters of each scan (as stored in the DICOM header) were manually checked post hoc against standard settings to detect inconsistencies in the data acquisition. Throughout the data processing pipeline, intermediate data volumes, segmentations, and parameter maps were systematically checked visually, especially to detect misregistration or erroneous scaling of quantitative maps.

### 2.4 Estimation of parameter maps

The MPM data were processed using a customized version of the hMRI‐toolbox (Tabelow et al., 2019), based on versions v0.1.1‐beta and v0.1.2‐beta, (available at https://github.com/tleutritz-cbs/hMRI-Toolbox/tree/BaudrexelRFspoiling) within the SPM12 framework (revision 7357; FIL, London, UK; RRID: SCR_007037) in MATLAB (version R2017b; Mathworks, Natick, MA; RRID: SCR_001622) on GNU/Linux computers with x86_64 architecture.

The main processing steps included the data conversion, calculation of quantitative maps, and reproducibility analyses. A collection of scripts encompassing all the following steps and a simplified ROI analysis within one subject is available on Github (https://github.com/tleutritz-cbs/MPM_quality).

DICOM images were first converted to NIfTI volumes using the hMRI‐toolbox converter and a comprehensive set of meta‐data in JSON files were stored for further processing (Tabelow et al., 2019). The Philips DICOM images were converted to NIfTI by applying scaling factors available from private tags, to enable quantitative evaluation of the data (Chenevert et al., 2014). The DICOM converter within the hMRI‐toolbox and within SPM12 (since version r7487) was adapted to take these scaling factors into account. Alternatively, Philips scanners have the opportunity to save data proportional to the MR signal within DICOMs as well. This could be achieved with a special setting within the reconstruction parameters (Density of Transverse Ordered Spins [DOTS]), but needs manual intervention for each session in “Gyrotest” mode. Unfortunately, this mode and the respective settings are not applicable within clinical trials. Furthermore, because the typical signal intensities of the Philips data differed widely from the default dynamic range threshold settings for the hMRI‐toolbox (originally designed for use with Siemens data), the settings for processing Philips data were changed as follows: MT‐threshold = 15 and A‐threshold = 10^8^. Customized parameter files with these modifications were loaded before further processing with the “Configure toolbox” module of the hMRI‐toolbox (Tabelow et al., 2019). The deletion of intermediate processing files (“cleanup”) was switched off. This enabled visual checks throughout the pipeline and the reuse of tissue segmentations for quality measures.

For optimal segmentation and registration of volumes, we first applied auto‐realignment as implemented in the hMRI‐toolbox (Tabelow et al., 2019). The first MT‐weighted echo was aligned to the PD‐weighted canonical template within SPM. Additional masking was applied to avoid segmentation issues due to noise outside the head. The masking was based on a python script for quality assessment within the Connectome project (Alfaro‐Almagro et al., 2018).

R1, PD, R2*, and MT maps were estimated from the multi‐echo data in combination with the B1^+^ and B1^−^ measurements using the “Create hMRI maps module” in the hMRI toolbox (Papp et al., 2016; Tabelow et al., 2019), based on the rational approximation of the signal equation presented by Helms, Dathe, and Dechent (2008). The R2* maps were calculated based on all echoes acquired across all contrasts using the ESTATICS estimation scheme (Weiskopf, Callaghan, Josephs, Lutti, & Mohammadi, 2014). To correct for R2*‐ and the remaining B1^−^‐related bias in the PD maps, the signal was extrapolated to TE = 0 (Ellerbrock & Mohammadi, 2018) and receive sensitivities were additionally corrected using a data based bias estimation according to UNICORT (Tabelow et al., 2019; Weiskopf et al., 2011). At one site (BSL), the B1^−^ maps suffered from excessive noise levels and could not be used for the MPM estimation. They had inadvertently been acquired with too high of a flip angle. Instead, B1^−^ was solely estimated using a data driven method based on unified segmentation and bias field correction within SPM (Ashburner & Friston, 2005; Tabelow et al., 2019). This is similar to UNICORT for B1^+^ correction (Tabelow et al., 2019; Weiskopf et al., 2011), but applied to PD maps only. All PD maps were calibrated to a value of 69 p.u. in WM (exceeding tissue probability of 95%) according to Tofts (2003).

To account for imperfect RF spoiling, we applied a voxel‐wise correction to the applied flip angles (after correction for B1^+/−^ inhomogenieties) depending on the applied phase increment (Table 2) according to the polynomial coefficients reported by Simon Baudrexel, Nöth, Schüre, and Deichmann (2017). The implementation of the method by Baudrexel et al. is available on github.com within a branch of the hMRI‐toolbox: https://codeload.github.com/tleutritz-cbs/hMRI-toolbox/zip/BaudrexelRFspoiling

## 3 HARMONIZATION OF MT MAPS

While the semi‐quantitative MT maps are largely insensitive to variations in local R1 values and B1^+^ fields (Callaghan et al., 2014; Helms, Dathe, & Dechent, 2008), they depend on the MT pulse used in the sequence (Table 2). The product sequences did not allow the user to precisely control the characteristics of the MT saturation pulses, thus a rescaling of MT maps was implemented to harmonize MT maps across manufacturers. The proposed harmonization also accounts for systematic differences in TR and measured R1 due to incidental MT by the excitation pulse (Olsson, Wirestam, Lätt, & Helms, 2020).

The estimated MT values (MT_orig_) from Philips scanners were linearly scaled to minimize the difference with the target MT values across pixels in brain tissue (ZH site arbitrarily served as reference):

(1) $$MT_{\text{scaled}}=a\;MT_{\text{orig}}+b$$

with two empirical parameters *a* and *b*, accounting for (a) the transferred saturation that is mainly driven by the saturation of the bound pool (i.e., power of the saturation pulse) and (b) a shift by direct saturation of the free water pool observed at frequency offsets <2 kHz (Helms, Dathe, & Dechent, 2008). Restriction to brain voxels was achieved by using gray matter (GM) and white matter (WM) masks determined by SPM unified segmentation (Ashburner & Friston, 2005) of the reference maps (ZH site). The GM/WM tissue probability masks were then set to a threshold of 99% to increase specificity to brain voxels. Cerebrospinal fluid (CSF) was explicitly excluded from the fitting procedure because it can exhibit direct saturation (offset) effects that would likely differ from tissue due to a much longer T2 and the absence of MT. In order to preserve the overall contrast, combined GM and WM masks were used for fitting. The resulting individual fitting parameters, over all subjects and scans from Philips sites, were used to estimate fixed scaling constants (*a*, *b*) by calculating the median of all fitted values. These two fixed parameters were then applied according to Equation (1) to all MT maps from Philips sites.

### 3.1 Analysis of inter‐ and intra‐site reproducibility

To determine intra‐ and inter‐site reproducibility of the MPM metrics, coefficients of variance (CoV) within and between sites were calculated voxel‐wise for each parameter map. To assess systematic bias, mean parameter values were additionally compared between sites. For the voxel‐based analysis, all quantitative maps were warped into common MNI space using DARTEL (Ashburner, 2007) as implemented within the hMRI toolbox. All subject data (including scan and rescan) from all sites was used to create the DARTEL template.

The intra‐site CoV was determined voxel‐wise as the *SD* (σ_intra_) of the quantitative maps estimated from scan and rescan data over the mean (μ_intra_) of both maps at a single site:

(2) $${\mathrm{CoV}}_{\text{intra}}\;\left(\mathrm{vx},\text{site},\text{subj},\mathrm{MPM}\right)=\sigma_{\text{intra}}/\mu_{\text{intra}}$$

with vx being the voxel number, site being the site where the data were acquired, subj being the subject identifier, and MPM being the mapped quantitative parameter. This represents the precision of MPM metrics within the same subject and site.

The inter‐site CoV was determined voxel‐wise as the *SD* (σ_intra_) over the mean (μ_intra_) across all scans for a specific subject, comprising bias observed at individual sites:

(3) $${\mathrm{CoV}}_{\text{inter}}\left(\mathrm{vx},\text{subj},\mathrm{MPM}\right)=\sigma_{\text{inter}}/\mu_{\text{inter}}$$

The site‐specific relative bias Δ was defined as the voxel‐wise ratio between μ_intra_ of the respective site and the mean of all μ_intra_ across all sites:

(4) $$\Delta \left(\mathrm{vx},\text{site},\text{subj},\mathrm{MPM}\right)=\mu_{\text{intra}}/\text{mean}\_\text{across}\_\text{sites}\left(\mu_{\text{intra}}\right)–1$$

Tissue probability maps for GM and WM from both (scan and rescan) MT maps were averaged per subject across all sites in order to provide a unified mask across all sites for better inter‐site comparison. These averages were then set to a threshold of 95% tissue probability, to serve as GM or WM tissue masks, respectively. Additional ROIs were extracted from FSL (version 5.0.9; FMRIB, Oxford, UK; RRID: SCR_002823) brain atlases (Desikan et al., 2006; Hua et al., 2008). The following ROIs were chosen because reference values were available (Weiskopf et al., 2013) or because significant pathological changes due to spinal cord injury have previously been observed within these ROIs (Freund et al., 2013; Freund, Rothwell, Craggs, Thompson, & Bestmann, 2011; Grabher et al., 2015; Villiger et al., 2015): caudate nucleus (CN), corpus callosum (CC), GM and WM of thalamus and cerebellum, cerebral peduncles, corticospinal tract (CST), hippocampi, as well as primary sensory (S1) and motor (M1) cortices, respectively. GM and WM masks and the additional ROIs (conjunct with the GM/WM mask, respectively) were used in the further ROI analysis.

The CoV_intra_ and CoV_inter_ for a specific ROI was determined by calculating the root‐mean‐square (RMS) value of the respective CoV measure across all *N* voxels within the ROI as follows:

(5) $$x_{\mathrm{RMS}}=\sqrt{\sum_{}^{}\left({x_{vx}}^{2}\;\right)/N}$$

Analogously, summary CoV values across sites were also determined by the RMS across sites. The systematic bias for a specific site was determined by calculating the RMS value of the bias Δ across all voxels within the GM and WM ROIs.

To assess systematic differences introduced by the acquisition protocols, which differed somewhat between manufacturers (e.g., different RF pulses or spoiling characteristics), the MPM data were reordered into three different groups: (a) data from all Siemens sites; (b) all Siemens data excluding the data from the BSL site, due to poor quality of B1^−^ maps; (c) data from all Philips sites. For easier assessment of these three groups, summary measures of CoV_intra_, CoV_inter_, and bias Δ were calculated as the RMS value across the GM and WM masks, subjects and sites, respectively.

## 4 RESULTS

### 4.1 Data quality control

The DICOM header consistency check found the following minor deviations from the planned acquisition protocols. A single data set at site HD was acquired with minor TE differences (< 4.1%), which were corrected by MPM estimation procedures (i.e., estimation at TE = 0). In addition, partial Fourier was set to 6/8 instead of 1 for low‐resolution scans for B1^−^ in the same data set, which also occurred occasionally at other sites (NOT, ZH). This may have impacted the effective resolution and signal‐to‐noise ratio (SNR). However, this was not recognized as being detrimental to the B1^−^ mapping measurements. At one site (BSL), the B1^−^ maps suffered from excessive noise levels due to incorrect flip angle settings (23° instead of 6°). These data could not be used for the B1^−^ estimation. Instead, a data driven method was used (see above). A data set of a single subject was acquired with a 1.1 mm in‐plane resolution at one site (BCN). Moreover, for a single scan the parallel acquisition was switched off, which may have affected the effective resolution, SNR, and introduced motion artifacts due to the longer scan time.

Overall the protocol check demonstrated good adherence to the planned acquisition protocol (only about 2.4% of the approximately 12,000 checked acquisition parameters differed).

The visual checks of intermediate processing results were used to optimize the processing pipeline, for example, introducing realignment and head masking as described in the methods.

### 4.2 Harmonization of MT maps

**FIGURE 1:**
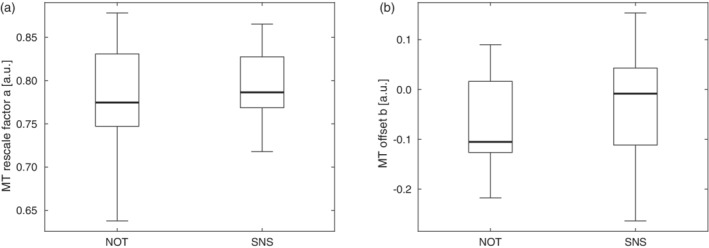
Range of fitted parameter values across subjects for MT harmonization of Philips scanner data: (a) scaling factor a, and (b) offset b. Box = interquartile range; bold horizontal line = median, feathers = data range of values across subjects

**FIGURE 2:**
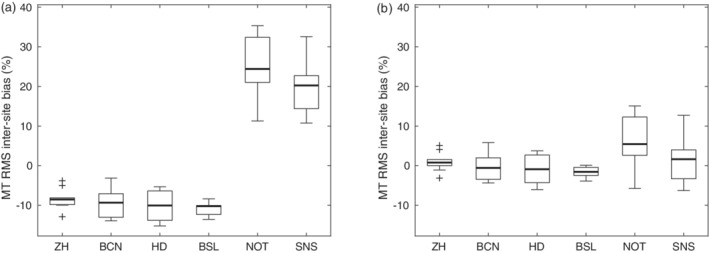
Relative inter‐site bias of the MT values across subjects per site (a) without and (b) with rescaling of MT values. Box = interquartile range; bold horizontal line = median, feathers = data range of values across subjects. Cross = outlier, which exceeds the range of (q1–1.5 × [q3−q1]; q3 + 1.5 × [q3−q1]), where q1 and q3 are the 25th and 75th percentiles of the sample data, respectively

The MT values obtained on the Philips scanners were harmonized using a linear model (Equation (1)). The coefficient of determination R^2^ was in the range of 0.81–0.91 for fitting Equation (1) across GM/WM ROIs. Fitting parameters varied between subjects and sites (Figure 1). The median of the scaling factor *a* was *a* = 0.7895 and for the offset *b* = −0.0807. Rescaling of the Philips data using these two fixed scaling factors reduced the inter‐site bias Δ after harmonization (Figure 2).

### 4.3 Inter‐ and intra‐site reproducibility and relative bias

**FIGURE 3:**
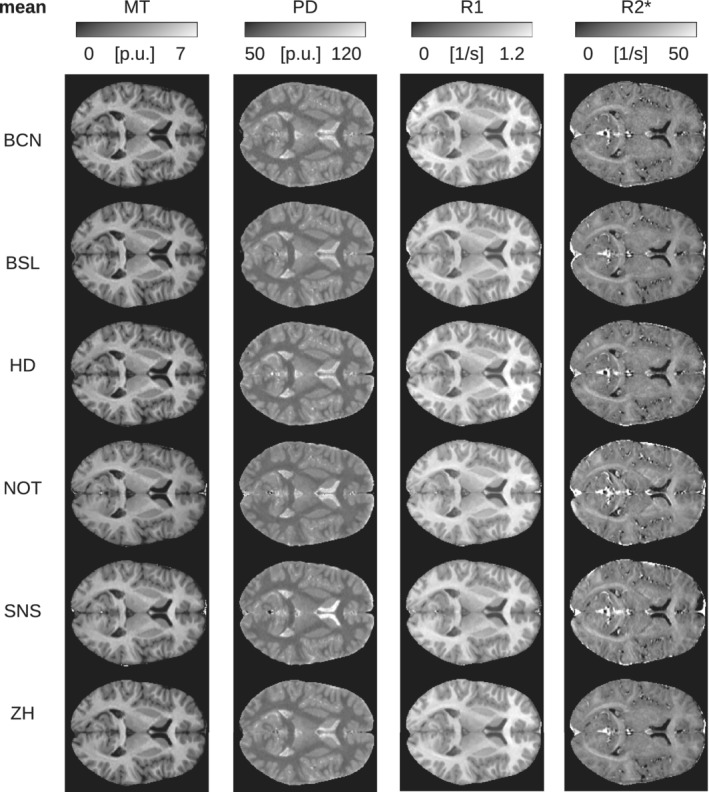
Mean of parameter maps shown for subject no. 5 scanned at all sites (axial slice through the center of the brain). The mean was calculated across the scan and rescan measurements

**FIGURE 4:**
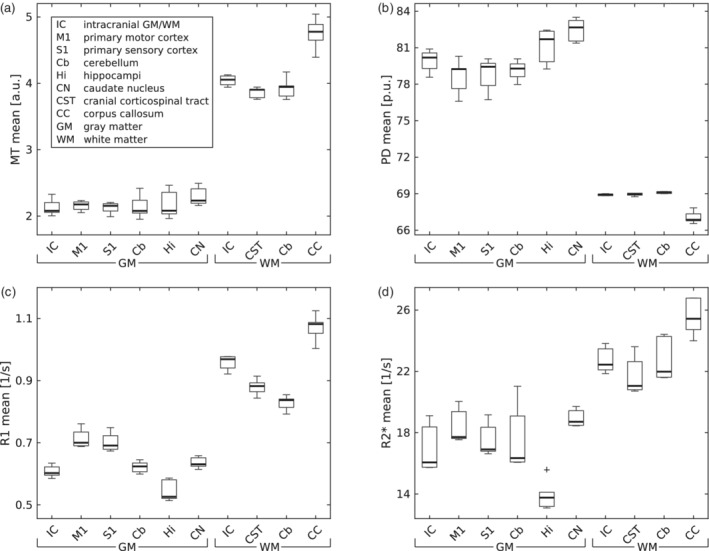
Distribution of (a) MT, (b) PD, (c) R1, and (d) R2* mean values in regions of interest (ROI) across subjects and sites. Please note that PD values are scaled to 69 p.u. for the mean WM value by the PD map estimation process (Tabelow et al., 2019; Tofts, 2003). Box = interquartile range; bold horizontal line = median, feathers = data range of values across subjects. Cross = outlier, which exceeds the range of (q1–1.5 × [q3−q1]; q3 + 1.5 × [q3−q1]), where q1 and q3 are the 25th and 75th percentiles of the sample data, respectively

**FIGURE 5:**
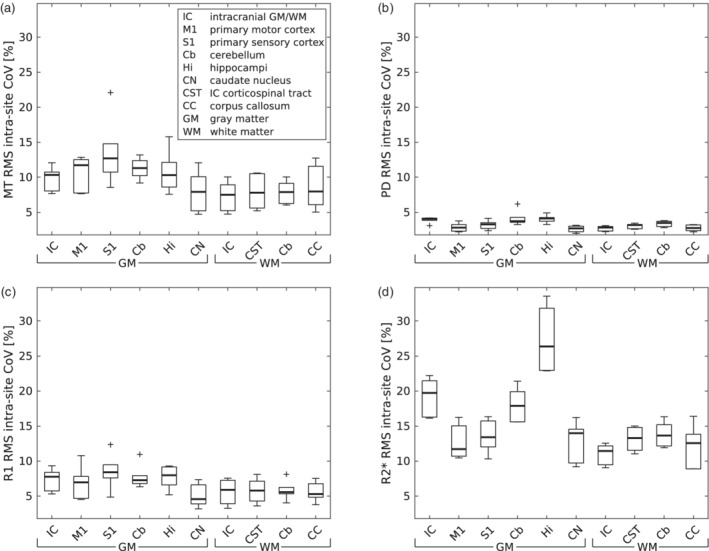
Distribution of (a) MT, (b) PD, (c) R1 and (d) R2* intra‐site CoV in regions of interest (ROI) across subjects within each site. Box = interquartile range; bold horizontal line = median, feathers = data range of values across subjects. Cross = outlier, which exceeds the range of (q1–1.5 × [q3−q1]; q3 + 1.5 × [q3−q1]), where q1 and q3 are the 25th and 75th percentiles of the sample data, respectively

**FIGURE 6:**
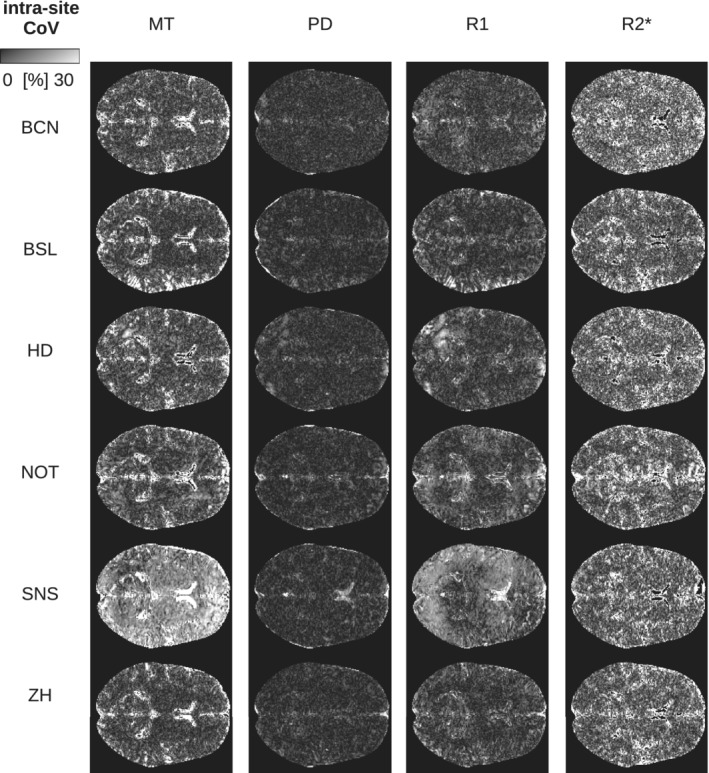
Intra‐site CoV of parameter maps shown for Subject 5 scanned at all sites (axial slice through the center of the brain). The intra‐site CoV was calculated across the scan and rescan measurements

**FIGURE 7:**
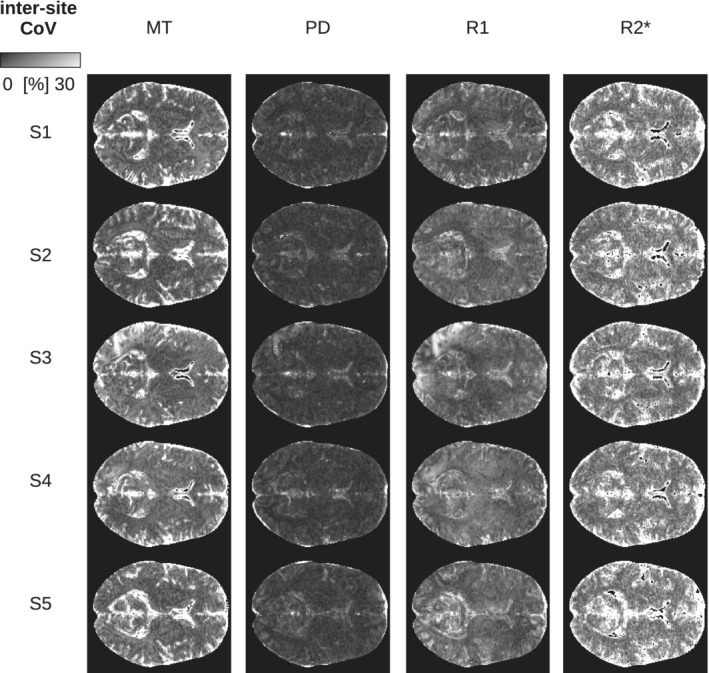
Inter‐site CoV of parameter maps shown for all subjects scanned at all sites (axial slice through the center of the brain). The inter‐site CoV was calculated over all six sites within the study

**FIGURE 8:**
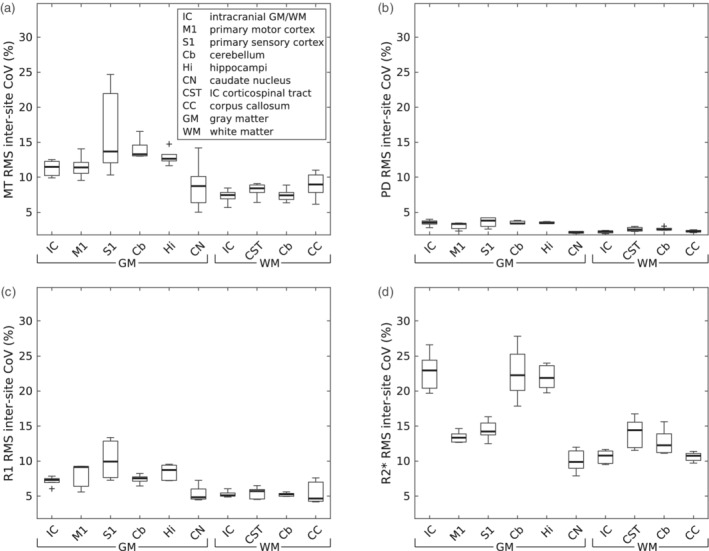
Distribution of (a) MT, (b) PD, (c) R1, and (d) R2* inter‐site CoV in regions of interest (ROI) across subjects and sites. Box = interquartile range; bold horizontal line = median, feathers = data range of values across subjects. Cross = outlier, which exceeds the range of (q1–1.5 × [q3−q1]; q3 + 1.5 × [q3−q1]), where q1 and q3 are the 25th and 75th percentiles of the sample data, respectively

**FIGURE 9:**
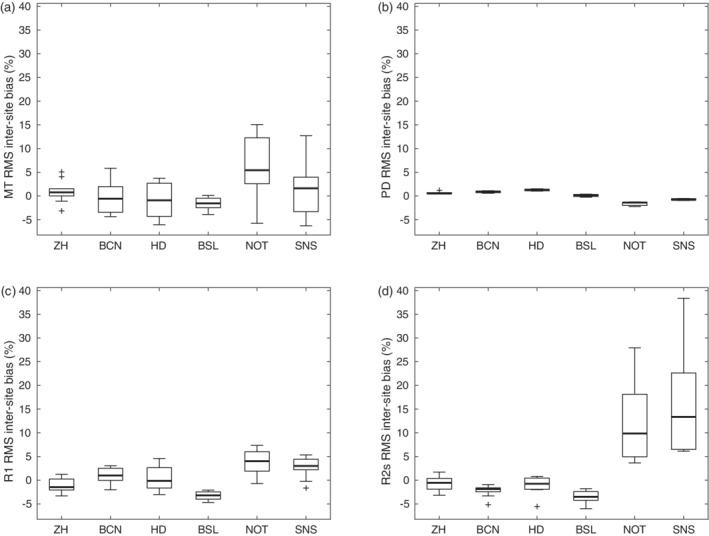
Distribution of the relative bias of (a) MT, (b) PD, (c) R1, and (d) R2* across GM/WM ROIs and subjects for each site. Box = interquartile range; bold horizontal line = median, feathers = data range of values across subjects. Cross = outlier, which exceeds the range of (q1–1.5 × [q3−q1]; q3 + 1.5 × [q3−q1]), where q1 and q3 are the 25th and 75th percentiles of the sample data, respectively

**FIGURE 10:**
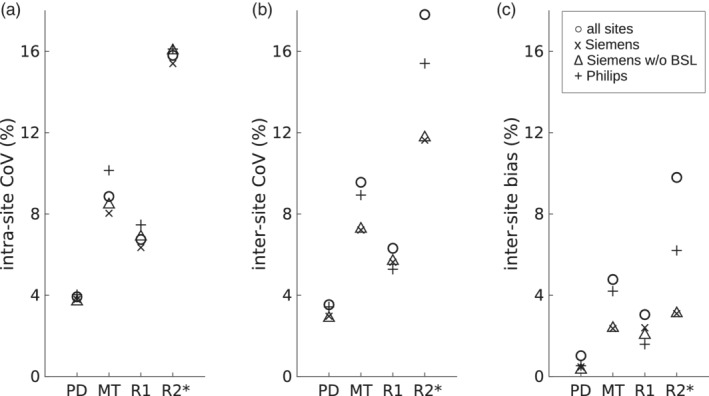
(a) Inter‐ and (b) intra‐site CoV, and (c) bias in a subgroup of sites, which is aggregated across all subjects/sites in the corresponding subgroup and the whole brain (GM and WM ROIs)

The MT, PD, R1, and R2* maps showed a distinct GM/WM contrast and different anatomical structures in the brain. For example, the cortex, cerebellum, midbrain structures, basal ganglia, thalamus, optic radiation, and ventricles could be visualized (see Figure 3 for maps of a representative subject; see Figure 4 for quantitative ROI analyses). The RMS average of intra‐site scan‐rescan CoV for GM and WM was between 8 and 10% for harmonized maps of MT, ~7% for R1, and 4% for PD. It was higher for R2* with a CoV of up to 16% (Figures 5 and 10; see Figure 6 for spatial distribution). The inter‐site CoV showed a pattern similar to the intra‐site CoV (Figure 6, Figure 7, Figure 5, and Figure 8), indicating a good alignment of measures across sites. Average inter‐site biases were between 0.8 and 4.8% for GM and WM for MT, PD, and R1 maps in the whole brain and rose to 9.8% for R2* maps (Figure 9 and Figure 10).

Next, the contribution of differences across the MRI acquisition protocols to the CoVs and bias were assessed by analyzing subgroups of sites and comparing these to the whole data set comprising all sites (Figure 10). Considered subgroups were (a) all Siemens data, (b) all Siemens data without BSL site data, because of the differing processing schemes for B1^−^, and (c) all Philips data. Generally, the CoVs were in a similar range for all data from the different subgroups, except for the inter‐site CoV and bias of R2*. The highest CoVs were found for the R2* measures independent of the data subgroup. The intra‐site CoVs were slightly increased for the Philips subgroup (c) data set.

## 5 DISCUSSION

We implemented and compared quantitative multiparameter mapping protocols based on product pulse sequences from two different MRI manufacturers within a traveling heads study. Protocols were designed to achieve a high isotropic resolution close to 1 mm and total acquisition times of ~20 min, making them suitable for use in clinical trials targeting specific anatomical and microstructural metrics.

### 5.1 Accuracy of quantitative maps

**FIGURE 11:**
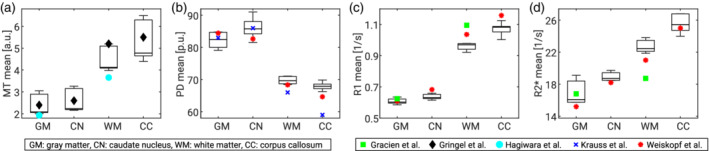
Mean values of parameter maps across subjects and sites for (a) MT, (b) PD, (c) R1, and (d) R2*. ROIs: Intracranial gray (GM) and white matter (WM), a sphere in the head of caudate nucleus (CN), a sphere in the corpus callosum (CC). Reference values marked by symbols (Gracien et al., 2019; Gringel et al., 2009; Hagiwara et al., 2018; Krauss, Gunnarsson, Andersson, & Thunberg, 2015; Weiskopf et al., 2013)

The mean values of MT, PD, R1, and R2* (Figure 11) were generally in line with values published by Gracien et al. (2019); Gringel et al. (2009); Hagiwara et al. (2018); Krauss et al. (2015); Weiskopf et al. (2013). Relaxation (R1, R2*) values deviated by 0.3–10.9% from the values reported in Weiskopf et al. (2013) in GM, WM, caudate nucleus (CN), and the corpus callosum (CC). PD values differed by 3.7–13.6% from those reported in Krauss et al. (2015), considering the same ROIs. For R1, and R2* we achieved deviations between 0.3 and 21.2%, and between 1.5 and 3.8% for PD compared with Gracien et al. (2019), taking only GM and WM mean values into account. MT values differed between 11 and 22% from values reported in Gringel et al. (2009), whereas the deviation was only about 10% from Hagiwara et al. (2018). Both reference studies (Gringel et al., 2009; Hagiwara et al., 2018) used pulse settings comparable to those applied here.

Quantitative PD and R1 maps generally rely on B1^+/−^ field correction, which was carried out with vendor sequences in this study, compared with reference studies. No direct comparison in regard to the actual accuracy of the applied sequences with custom‐made sequences was attempted. Any degree of erroneous field correction influences R1 maps by a factor of two, due to quadratic dependence on the actual flip angle. For example, the custom‐made B1 mapping applied in the study by Weiskopf et al. (2013) is accurate with a total error of less than ca. 3% (Lutti et al., 2010), contributing to an error rate of about 6% in R1 maps. In addition, the correction of imperfect RF spoiling relies on accurate flip angles and will enhance errors as well, which should be accounted for in the series of error propagation. The calibration of PD maps to a fixed value of 69 p. u. in WM (Tofts, 2003) might introduce a bias in these maps, not reflecting pathologic changes in WM. Moreover, it is known that R1 and PD values are affected by inadvertent magnetization transfer effects, which depend on the specifics of the RF pulse configuration and power (Teixeira, Malik, & Hajnal, 2019).

Closed‐source filter settings at Philips sites could not be controlled and thus might also have influenced the SNR of the data. Approximation to a TE of 0 reduced R2* biases, but might have introduced additional noise sources. Furthermore, residual deviations may have been driven by methodological differences (Stikov et al., 2015) or instrumental differences but also inter‐individual biological variation (Figure 11), since different cohorts were studied. Additionally, the approaches varied in B1^+/−^ field correction methods, treatment of incomplete RF spoiling biases, and different aspects of data processing, which may explain some differences to reference values. We did not account for the slight differences in field strengths between the two vendors (2.89 T for Siemens, 3.00 T for Philips MRI), which may have led to a small bias of ~1.4% in T1 relaxation (Rooney et al., 2007).

However, this study went beyond previous multicenter studies using quantitative mapping (Deoni et al., 2008; Gracien et al., 2019; Lee, Callaghan, Acosta‐Cabronero, Lutti, & Nagy, 2019; Weiskopf et al., 2013) by including six sites, two different manufacturers, and four different MRI scanner models. By including diverse hardware and software, this study captures the typical situation in multicenter clinical trials better and provides a more realistic reference for the performance of MPM in multicenter studies using widely available product sequences and methods.

### 5.2 Handling of protocol deviations

Inadvertent deviations from the standard protocols may occur in large complex imaging studies. Here, they included slight variations in echo times or spatial encoding settings. The flexibility of the hMRI processing toolbox and the intrinsic advantages of quantitative MRI allowed for a principled correction. For example, differing TE values were addressed by the fitting of R2* rather than relying on R2*‐weighted images and also by extrapolating the signal to TE = 0 for calculation of MT, R1, and PD maps. Other deviations mainly influenced the acquisition time and only to a certain degree the resulting measurements (e.g., differing signal‐to‐noise ratio). To help avoid incorrect settings of protocol parameters in upcoming multicenter studies, our traveling heads study was used to optimize standard operating procedures. The CoVs may be further reduced by selecting a subgroup of Siemens sites (excluding the data acquired with poor B1^−^ maps due to a fault in the flip angle settings and required adjustments of the processing).

### 5.3 Intra‐ and inter‐site CoV and bias

The proposed MPM acquisition achieved a high inter‐site comparability with a low inter‐site bias of less than 5% in the quantitative maps. Similarly, the intra‐ and inter‐site CoV were in a range of 5–10% for R1 and MT maps. Thus, the observed CoV was ~3 times lower than the trauma‐related effect sizes shown in longitudinal studies of spinal cord injury 12 months after injury, which range from 17 to 20% for R1, and 14% for MT (Grabher et al., 2015). Hence, the presented MPM protocol is expected to reliably detect and characterize the trauma‐related effects in longitudinal multicenter studies. Despite the harmonization of MT values across vendor platforms the intra‐ and inter‐site CoVs remained slightly elevated for the subgroup of Philips sites (Figure 10), also influencing the overall results across all sites (Figure 5 and Figure 8). This might also be related to the actual implementation of RF spoiling correction, which is equally applied to T1‐, PD‐, and MT‐weighted multi‐echo data. Nevertheless, the inter‐site bias for all sites was decreased from 15.7 to 4.8% as a result of MT harmonization. Considering the calibration of PD maps to 69 p. u. in WM, the low CoVs and bias might be underestimated.

A higher inter‐site CoV of 11.6–17.8% was observed for R2* maps, which was partly driven by the higher intra‐site CoV for R2* maps and systematic differences between the two manufacturers (Figure 10). This includes field strength and maximum TE (14.76 ms; Table 2), which is not optimal for estimating R2* in GM or WM. We also attribute the intra‐ and inter‐site differences in R2* to the relatively poor reproducibility and performance of shimming routines. Further studies should be performed to elucidate the vendor differences observed in our study, which should also include simulation of vendor sequences. Another source of variability of R2* might arise from magnetic field inhomogeneities, which could be corrected with the approach by S. Baudrexel et al. (2009).

## 6 CONSIDERATIONS

The current implementation of spoiling corrections, based on the vendor's spoiling schemes (Simon Baudrexel et al., 2017) within the hMRI‐toolbox, is still limited to vendor specific phase increments and might not be applied to the MT‐weighted multi‐echo data, where different spoiling schemes are applied.

The differences in MT pulses between manufacturers resulted in ~20% difference in MT saturation values, which were harmonized in the post‐processing by a linear rescaling of the MT values. This reduced the inter‐site bias considerably (Figure 2). This traveling heads study may serve as a reference for the rescaling of MT values in future multi‐vendor studies. Because of rescaling, MT values may not be comparable to previous studies, for example, due to differences between custom‐made sequences with optimized MT pulse scheme (Weiskopf et al., 2013), and vendor‐based sequences and MT pulses (Table 2).

The small sample size of five volunteers in a narrow age range may not represent the general population and its variability. Thus, care should be taken when extrapolating these results to different patient or subject groups, for example, populations of elderly patients. However, we believe that most of the study characteristics are fundamental and will be only modulated by the population studied. For instance, larger head motion will lead to general increases in CoV, which will add to the characteristics described here.

Due to the short time gap of 2 hr between the measurements, the scan‐rescan experiment mimics, but cannot fully capture, the variance components in long term longitudinal studies (e.g., instrumental deterioration, long term physiological fluctuations, hardware/software changes). Thus, we would consider the intra‐site CoV as an approximation and a lower limit of variability in longitudinal studies.

We only used vendor‐based sequences to make the MPM approach widely accessible. However, we included specialized vendor sequences for acquisition of the B1^+^ mapping reference data, which may depend on the software baseline. In case of the B1^+^ field mapping on the Philips platform, this required clinical science keys (CSK) (option 047). Alternatives for Philips scanners without this CSK would be the use of vendor sequences for double angle B1^+^ mapping methods (Boudreau et al., 2017) or the use of data driven postprocessing correction methods such as UNICORT (Weiskopf et al., 2011)

The correction of biases related to B1^−^ field inhomogoneities consisted of two main steps. Sensitivity maps acquired between all multi‐echo gradient echo sequences were used to correct for apparent sensitivity changes due to head motion between the acquisitions, that is, motion between different contrasts within the acquisition scheme of MPM (Papp et al., 2016). Since the method assumes the body coil RF receive sensitivity field to be uniform for calibration, the PD maps will be affected by any body coil sensitivity inhomogeneities. Thus, in the second step an additional correction was applied using a data driven bias estimation analogous to UNICORT and as implemented in the hMRI‐toolbox (Tabelow et al., 2019; Weiskopf et al., 2011). This reduced the inter‐ and intra‐site CoV further (data not shown)

Additionally, we improved the standard processing pipeline in the hMRI‐toolbox (Tabelow et al., 2019) by applying a head mask to reduce segmentation errors, as well as an additional implementation of correction for imperfect RF spoiling (Simon Baudrexel et al., 2017), reducing measurement biases.

## 7 CONCLUSIONS

This study investigated scan‐rescan and inter‐site reproducibility of the multiparameter mapping (MPM) approach implemented, at 3 T MRI, with Philips and Siemens vendor sequences. The aim of the study was to generally enable and additionally improve the comparability of multicenter studies. The 1 mm resolution MPM maps showed high repeatability and comparability across different testing sites. The measurements were comparable, as reflected by a low inter‐site bias (below 5%) and highly reproducible for quantitative maps of MT, R1, and PD. Intra‐site coefficients of variation for these measures ranged between 4 and 10% and up to 18% for R2* maps. Quantitative MRI parameters were in good agreement with previously reported studies (Weiskopf et al., 2013), with small deviations on the order of 0.3–10.9%. Since we used only vendor product sequences for the data acquisition, and the open source hMRI‐toolbox (www.hMRI.info; Tabelow et al. (2019)) for processing, the approach can be readily applied in quantitative MRI single‐ and multisite studies.

## CONFLICT OF INTEREST

The Max Planck Institute for Human Cognitive and Brain Sciences and Wellcome Centre for Human Neuroimaging have institutional research agreements with Siemens Healthcare. NW was a speaker at an event organized by Siemens Healthcare and was reimbursed for the associated travel expenses.

## Supporting information


**Appendix** S1. Supporting Information

## Data Availability

The anonymized data that support the findings of this study are available from the corresponding author upon reasonable request. However, the personal, non‐anonymized MRI data cannot be made available, since their sharing is constrained by ethical and European GDPR regulations.

## References

[hbm25122-bib-0001] Alfaro‐Almagro, F. , Jenkinson, M. , Bangerter, N. K. , Andersson, J. L. R. , Griffanti, L. , Douaud, G. , … Smith, S. M. (2018). Image processing and quality control for the first 10,000 brain imaging datasets from UKbiobank. NeuroImage, 166, 400–424. 10.1016/j.neuroimage.2017.10.034 29079522 PMC5770339

[hbm25122-bib-0002] Ashburner, J. (2007). A fast diffeomorphic image registration algorithm. NeuroImage, 38(1), 95–113. 10.1016/j.neuroimage.2007.07.007 17761438

[hbm25122-bib-0003] Ashburner, J. , & Friston, K. J. (2005). Unified segmentation. NeuroImage, 26(3), 839–851. 10.1016/j.neuroimage.2005.02.018 15955494

[hbm25122-bib-0004] Baudrexel, S. , Nöth, U. , Schüre, J.‐R. , & Deichmann, R. (2017). T1 mapping with the variable flip angle technique: A simple correction for insufficient spoiling of transverse magnetization. Magnetic Resonance in Medicine, 79(6), 3082–3092. 10.1002/mrm.26979 29052267

[hbm25122-bib-0005] Baudrexel, S. , Volz, S. , Preibisch, C. , Klein, J. C. , Steinmetz, H. , Hilker, R. , & Deichmann, R. (2009). Rapid single‐scan T2*‐mapping using exponential excitation pulses and image‐based correction for linear background gradients. Magnetic Resonance in Medicine, 62(1), 263–268. 10.1002/mrm.21971 19353655

[hbm25122-bib-0006] Boudreau, M. , Tardif, C. L. , Stikov, N. , Sled, J. G. , Lee, W. , & Pike, G. B. (2017). B1 mapping for bias‐correction in quantitative T 1 imaging of the brain at 3T using standard pulse sequences. Journal of Magnetic Resonance Imaging, 46(6), 1673–1682. 10.1002/jmri.25692 28301086

[hbm25122-bib-0007] Callaghan, M. F. , Freund, P. , Draganski, B. , Anderson, E. , Cappelletti, M. , Chowdhury, R. , … Weiskopf, N. (2014). Widespread age‐related differences in the human brain microstructure revealed by quantitative magnetic resonance imaging. Neurobiology of Aging, 35(8), 1862–1872. 10.1016/j.neurobiolaging.2014.02.008 24656835 PMC4024196

[hbm25122-bib-0008] Cercignani, M. , Dowell, N. G. , & Tofts, P. (2018). Quantitative MRI of the brain: Principles of physical measurement, Boca Raton, FL: CRC Press.

[hbm25122-bib-0009] Chenevert, T. L. , Malyarenko, D. I. , Newitt, D. , Li, X. , Jayatilake, M. , Tudorica, A. , … Ross, B. D. (2014). Errors in quantitative image analysis due to platform‐dependent image scaling. Translational Oncology, 7(1), 65–71. 10.1593/tlo.13811 24772209 PMC3998685

[hbm25122-bib-0010] Chung, S. , Kim, D. , Breton, E. , & Axel, L. (2010). Rapid B1+ mapping using a preconditioning RF pulse with TurboFLASH readout. Magnetic Resonance in Medicine, 64(2), 439–446. 10.1002/mrm.22423 20665788 PMC2929762

[hbm25122-bib-0011] Dathe, H. , & Helms, G. (2010). Exact algebraization of the signal equation of spoiled gradient echo MRI. Physics in Medicine and Biology, 55(15), 4231–4245. 10.1088/0031-9155/55/15/003 20616401

[hbm25122-bib-0012] Deoni, S. C. L. , Williams, S. C. R. , Jezzard, P. , Suckling, J. , Murphy, D. G. M. , & Jones, D. K. (2008). Standardized structural magnetic resonance imaging in multicentre studies using quantitative T1 and T2 imaging at 1.5 T. NeuroImage, 40(2), 662–671. 10.1016/j.neuroimage.2007.11.052 18221894

[hbm25122-bib-0013] Desikan, R. S. , Ségonne, F. , Fischl, B. , Quinn, B. T. , Dickerson, B. C. , Blacker, D. , … Killiany, R. J. (2006). An automated labeling system for subdividing the human cerebral cortex on MRI scans into gyral based regions of interest. NeuroImage, 31(3), 968–980. 10.1016/j.neuroimage.2006.01.021 16530430

[hbm25122-bib-0014] Edwards, L. , Kirilina, E. , Mohammadi, S. , & Weiskopf, N. (2018). Microstructural imaging of human neocortex in vivo. NeuroImage, 182, 184–206. 10.1016/j.neuroimage.2018.02.055 29588229

[hbm25122-bib-0015] Ellerbrock, I. , & Mohammadi, S. (2018). Four in vivo g‐ratio‐weighted imaging methods: Comparability and repeatability at the group level. Human Brain Mapping, 39(1), 24–41. 10.1002/hbm.23858 29091341 PMC6866374

[hbm25122-bib-0016] Filippi, M. , & Agosta, F. (2007). Magnetization transfer MRI in multiple sclerosis. Journal of Neuroimaging, 17, 22S–26S. 10.1111/j.1552-6569.2007.00132.x 17425730

[hbm25122-bib-0017] Freund, P. , Rothwell, J. , Craggs, M. , Thompson, A. J. , & Bestmann, S. (2011). Corticomotor representation to a human forearm muscle changes following cervical spinal cord injury. The European Journal of Neuroscience, 34(11), 1839–1846. 10.1111/j.1460-9568.2011.07895.x 22082003

[hbm25122-bib-0018] Freund, P. , Seif, M. , Weiskopf, N. , Friston, K. , Fehlings, M. G. , Thompson, A. J. , & Curt, A. (2019). MRI in traumatic spinal cord injury: From clinical assessment to neuroimaging biomarkers. The Lancet Neurology, 18(12), 1123–1135. 10.1016/S1474-4422(19)30138-3 31405713

[hbm25122-bib-0019] Freund, P. , Weiskopf, N. , Ashburner, J. , Wolf, K. , Sutter, R. , Altmann, D. R. , … Curt, A. (2013). MRI investigation of the sensorimotor cortex and the corticospinal tract after acute spinal cord injury: A prospective longitudinal study. Lancet Neurology, 12(9), 873–881. 10.1016/S1474-4422(13)70146-7 23827394 PMC3744750

[hbm25122-bib-0020] Grabher, P. , Callaghan, M. F. , Ashburner, J. , Weiskopf, N. , Thompson, A. J. , Curt, A. , & Freund, P. (2015). Tracking sensory system atrophy and outcome prediction in spinal cord injury. Annals of Neurology, 78(5), 751–761. 10.1002/ana.24508 26290444 PMC4737098

[hbm25122-bib-0021] Gracien, R.‐M. , Maiworm, M. , Brüche, N. , Shrestha, M. , Nöth, U. , Hattingen, E. , … Deichmann, R. (2019). How stable is quantitative MRI? – Assessment of intra‐ and inter‐scanner‐model reproducibility using identical acquisition sequences and data analysis programs. NeuroImage, 207, 116364–116364. 10.1016/j.neuroimage.2019.116364 31740340

[hbm25122-bib-0022] Gringel, T. , Schulz‐Schaeffer, W. , Elolf, E. , Frolich, A. , Dechent, P. , & Helms, G. (2009). Optimized high‐resolution mapping of magnetization transfer (MT) at 3 tesla for direct visualization of substructures of the human thalamus in clinically feasible measurement time. Journal of Magnetic Resonance Imaging, 29(6), 1285–1292. 10.1002/jmri.21756 19472385

[hbm25122-bib-0023] Hagiwara, A. , Hori, M. , Kamagata, K. , Warntjes, M. , Matsuyoshi, D. , Nakazawa, M. , … Aoki, S. (2018). Myelin measurement: Comparison between simultaneous tissue Relaxometry, magnetization transfer saturation index, and T1w/T2w ratio methods. Scientific Reports, 8(1), 10554 10.1038/s41598-018-28852-6 30002497 PMC6043493

[hbm25122-bib-0024] Helms, G. , Dathe, H. , & Dechent, P. (2008). Quantitative FLASH MRI at 3T using a rational approximation of the Ernst equation. Magnetic Resonance in Medicine, 59(3), 667–672. 10.1002/mrm.21542 18306368

[hbm25122-bib-0025] Helms, G. , Dathe, H. , Kallenberg, K. , & Dechent, P. (2008). High‐resolution maps of magnetization transfer with inherent correction for RF inhomogeneity and T1 relaxation obtained from 3D FLASH MRI. Magnetic Resonance in Medicine, 60(6), 1396–1407. 10.1002/mrm.21732 19025906

[hbm25122-bib-0026] Helms, G. , Dathe, H. , Weiskopf, N. , & Dechent, P. (2011). Identification of signal bias in the variable flip angle method by linear display of the algebraic Ernst equation. Magnetic Resonance in Medicine, 66(3), 669–677. 10.1002/mrm.22849 21432900 PMC3193384

[hbm25122-bib-0027] Horsfield, M. A. , Barker, G. J. , Barkhof, F. , Miller, D. H. , Thompson, A. J. , & Filippi, M. (2003). Guidelines for using quantitative magnetization transfer magnetic resonance imaging for monitoring treatment of multiple sclerosis. Journal of Magnetic Resonance Imaging, 17(4), 389–397. 10.1002/jmri.10266 12655577

[hbm25122-bib-0028] Hua, K. , Zhang, J. , Wakana, S. , Jiang, H. , Li, X. , Reich, D. S. , … Mori, S. (2008). Tract probability maps in stereotaxic spaces: Analyses of white matter anatomy and tract‐specific quantification. NeuroImage, 39(1), 336–347. 10.1016/j.neuroimage.2007.07.053 17931890 PMC2724595

[hbm25122-bib-0029] Krauss, W. , Gunnarsson, M. , Andersson, T. , & Thunberg, P. (2015). Accuracy and reproducibility of a quantitative magnetic resonance imaging method for concurrent measurements of tissue relaxation times and proton density. Magnetic Resonance Imaging, 33(5), 584–591. 10.1016/j.mri.2015.02.013 25708264

[hbm25122-bib-0030] Kucher, K. , Johns, D. , Maier, D. , Abel, R. , Badke, A. , Baron, H. , … Curt, A. (2018). First‐in‐man Intrathecal application of neurite growth‐promoting anti‐nogo‐a antibodies in acute spinal cord injury. Neurorehabilitation and Neural Repair, 32(6–7), 578–589. 10.1177/1545968318776371 29869587

[hbm25122-bib-0031] Lee, Y. , Callaghan, M. F. , Acosta‐Cabronero, J. , Lutti, A. , & Nagy, Z. (2019). Establishing intra‐ and inter‐vendor reproducibility of T1 relaxation time measurements with 3T MRI. Magnetic Resonance in Medicine, 81(1), 454–465. 10.1002/mrm.27421 30159953

[hbm25122-bib-0032] Lutti, A. , Hutton, C. , Finsterbusch, J. , Helms, G. , & Weiskopf, N. (2010). Optimization and validation of methods for mapping of the radiofrequency transmit field at 3T. Magnetic Resonance in Medicine, 64(1), 229–238. 10.1002/mrm.22421 20572153 PMC3077518

[hbm25122-bib-0033] Mahajan, K. R. , & Ontaneda, D. (2017). The role of advanced magnetic resonance imaging techniques in multiple sclerosis clinical trials. Neurotherapeutics, 14(4), 905–923. 10.1007/s13311-017-0561-8 28770481 PMC5722766

[hbm25122-bib-0034] Olsson, H. A. , Wirestam, R. , Lätt, J. , & Helms, G. (2020). Reducing bias in dual flip angle T1‐mapping in human brain at 7T. Magnetic Resonance in Medicine, 84(3), 1347–1358. 10.1002/mrm.28206 32060952

[hbm25122-bib-0035] Papp, D. , Callaghan, M. F. , Meyer, H. , Buckley, C. , & Weiskopf, N. (2016). Correction of inter‐scan motion artifacts in quantitative R1 mapping by accounting for receive coil sensitivity effects. Magnetic Resonance in Medicine, 76(5), 1478–1485. 10.1002/mrm.26058 26608936 PMC5082493

[hbm25122-bib-0036] Rooney, W. D. , Johnson, G. , Li, X. , Cohen, E. R. , Kim, S.‐G. , Ugurbil, K. , & Springer, C. S. (2007). Magnetic field and tissue dependencies of human brain longitudinal 1H2O relaxation in vivo. Magnetic Resonance in Medicine, 57(2), 308–318. 10.1002/mrm.21122 17260370

[hbm25122-bib-0037] Seif, M. , Curt, A. , Thompson, A. J. , Grabher, P. , Weiskopf, N. , & Freund, P. (2018). Quantitative MRI of rostral spinal cord and brain regions is predictive of functional recovery in acute spinal cord injury. NeuroImage. Clinical, 20, 556–563. 10.1016/j.nicl.2018.08.026 30175042 PMC6115607

[hbm25122-bib-0038] Stikov, N. , Boudreau, M. , Levesque, I. R. , Tardif, C. L. , Barral, J. K. , & Pike, G. B. (2015). On the accuracy of T1 mapping: Searching for common ground. Magnetic Resonance in Medicine, 73(2), 514–522. 10.1002/mrm.25135 24578189

[hbm25122-bib-0039] Tabelow, K. , Balteau, E. , Ashburner, J. , Callaghan, M. F. , Draganski, B. , Helms, G. , … Mohammadi, S. (2019). hMRI ‐ ‐ A toolbox for quantitative MRI in neuroscience and clinical research. NeuroImage, 194, 191–210. 10.1016/j.neuroimage.2019.01.029 30677501 PMC6547054

[hbm25122-bib-0040] Teixeira, R. P. A. G. , Malik, S. J. , & Hajnal, J. V. (2019). Fast quantitative MRI using controlled saturation magnetization transfer. Magnetic Resonance in Medicine, 81(2), 907–920. 10.1002/mrm.27442 30257044 PMC6492254

[hbm25122-bib-0041] Tofts, P. S. (2003). Proton density of tissue water In Tofts P. S. (Ed.), Quantitative MRI of the brain: Measuring changes caused by disease (pp. 85–110). Chichester, UK: Wiley & Sons.

[hbm25122-bib-0042] Van Horn, J. D. , & Toga, A. W. (2009). Multisite neuroimaging trials. Current Opinion in Neurology, 22(4), 370–378. 10.1097/WCO.0b013e32832d92de 19506479 PMC2777976

[hbm25122-bib-0043] Villiger, M. , Grabher, P. , Hepp‐Reymond, M.‐C. , Kiper, D. , Curt, A. , Bolliger, M. , … Freund, P. (2015). Relationship between structural brainstem and brain plasticity and lower‐limb training in spinal cord injury: A longitudinal pilot study. Frontiers in Human Neuroscience, 9, 254–254. 10.3389/fnhum.2015.00254 25999842 PMC4420931

[hbm25122-bib-0044] Weiskopf, N. , Callaghan, M. F. , Josephs, O. , Lutti, A. , & Mohammadi, S. (2014). Estimating the apparent transverse relaxation time (R2[*]) from images with different contrasts (ESTATICS) reduces motion artifacts. Frontiers in Neuroscience, 8, 278–278. 10.3389/fnins.2014.00278 25309307 PMC4159978

[hbm25122-bib-0045] Weiskopf, N. , Lutti, A. , Helms, G. , Novak, M. , Ashburner, J. , & Hutton, C. (2011). Unified segmentation based correction of R1 brain maps for RF transmit field inhomogeneities (UNICORT). NeuroImage, 54(3), 2116–2124. 10.1016/j.neuroimage.2010.10.023 20965260 PMC3018573

[hbm25122-bib-0046] Weiskopf, N. , Mohammadi, S. , Lutti, A. , & Callaghan, M. F. (2015). Advances in MRI‐based computational neuroanatomy: From morphometry to in‐vivo histology. Current Opinion in Neurology, 28(4), 313–322. 10.1097/WCO.0000000000000222 26132532

[hbm25122-bib-0047] Weiskopf, N. , Suckling, J. , Williams, G. , Correia, M. M. , Inkster, B. , Tait, R. , … Lutti, A. (2013). Quantitative multi‐parameter mapping of R1, PD*, MT, and R2* at 3T: A multi‐center validation. Frontiers in Neuroscience, 7, 95 10.3389/fnins.2013.00095 23772204 PMC3677134

[hbm25122-bib-0048] Willcocks, R. J. , Rooney, W. D. , Triplett, W. T. , Forbes, S. C. , Lott, D. J. , Senesac, C. R. , … Vandenborne, K. (2016). Multicenter prospective longitudinal study of magnetic resonance biomarkers in a large duchenne muscular dystrophy cohort. Annals of Neurology, 79(4), 535–547. 10.1002/ana.24599 26891991 PMC4955760

[hbm25122-bib-0049] Yarnykh, V. L. (2007). Actual flip‐angle imaging in the pulsed steady state: A method for rapid three‐dimensional mapping of the transmitted radiofrequency field. Magnetic Resonance in Medicine, 57(1), 192–200. 10.1002/mrm.21120 17191242

[hbm25122-bib-0050] Yarnykh, V. L. (2010). Optimal radiofrequency and gradient spoiling for improved accuracy of T1 and B1 measurements using fast steady‐state techniques. Magnetic Resonance in Medicine, 63(6), 1610–1626. 10.1002/mrm.22394 20512865

[hbm25122-bib-0051] Ziegler, G. , Grabher, P. , Thompson, A. , Altmann, D. , Hupp, M. , Ashburner, J. , … Freund, P. (2018). Progressive neurodegeneration following spinal cord injury: Implications for clinical trials. Neurology, 90(14), e1257–e1266. 10.1212/WNL.0000000000005258 29514946 PMC5890610

