# Supplementary material for: Multiparameter mapping of relaxation (R1, R2*), proton density and magnetization transfer saturation at 3 T: A multicenter dual‐vendor reproducibility and repeatability study
Source: Hum Brain Mapp. 2020 Jul 8;41(15):4232–47. doi: 10.1002/hbm.25122 (PMC7502832; doi:10.1002/hbm.25122)
Supplement: Supplementary file 1 — Appendix S1. Supporting Information [file HBM-41-4232-s001.pdf]

This document contains the **Siemens 3T MRI** (pages 2-3) and **Philips 3T MRI** (pages 4-7) protocol settings, which are specific for the study protocol within the traveling heads study performed in scope of the NISCI trial (http://nisci-2020.eu/). To keep it shorter, we deleted parameters, which does not differ from standard settings (as far as we knew) and which are redundant within standard PDF printouts.

Here's the complete list of protocols to be set up and the acquisition scheme:

- rf_map (B1 mapping)
- RF_sens_head and RF_sens_body (RF sensitivity mapping)
- MPM_T1 (multi-echo MPM)
- MPM_PD (multi-echo MPM)
- MPM_MT (multi-echo MPM)

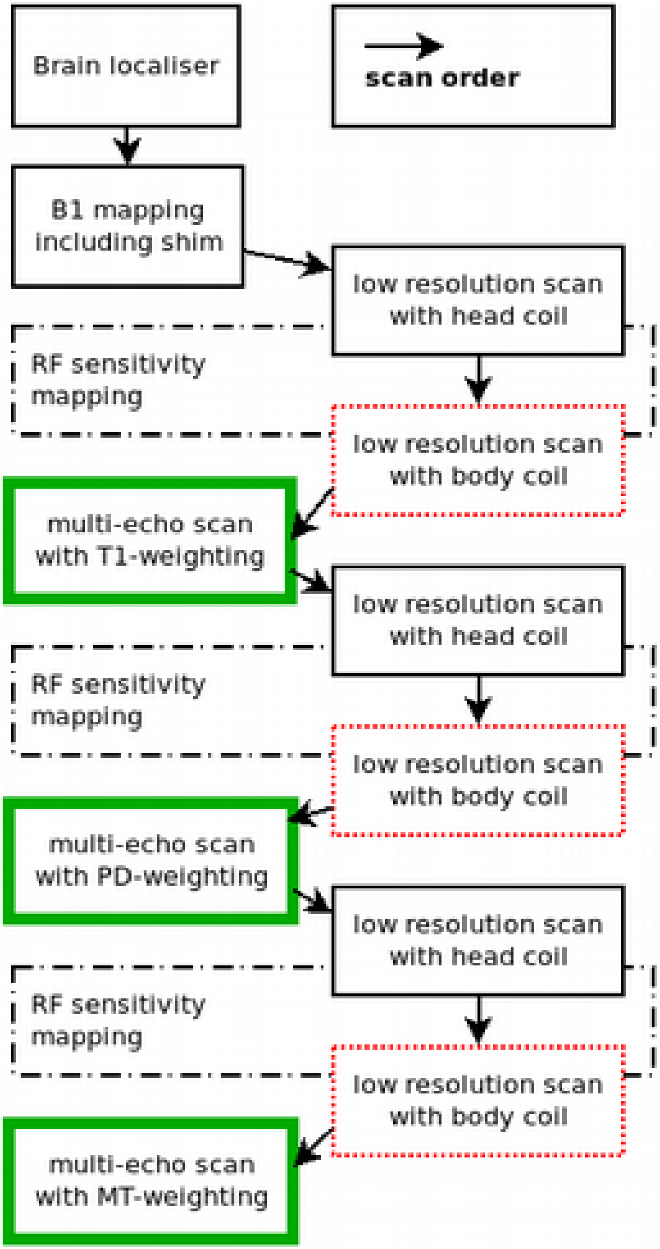

rf_map TA:2:14 Voxel size:4.0×4.0×5.0 mm

Source protocol: Siemens\SequenceRegion\ServiceSequences\DefaultProtocols\rf_map

Routine		Contrast	
Slices	18	Flip angle 1	90 deg
Dist. factor	100%	Measurements	1
Position	Isocenter	Resolution	
Orientation	Sagittal	Base resolution	64
Phase enc. dir.	A >> P	Phase resolution	100%
FoV read	256 mm	Image Filter	Off
FoV phase	100%	Distortion Corr.	Off
Slice thickness	5 mm	Prescan Normalize	Off
TR	2000 ms	Normalize	Off
TE 1	14 ms	B1 filter	Off
Averages	1	Raw filter	Off
Filter	None	Elliptical filter	Off
Coil elements	{head_neck}	System	
		Shim mode	Standard

RF_sens_head TA:9.8 s PAT:Off Voxel size:4.0×4.0×4.0 mm

Source protocol: Siemens\head\library_3D\t1_fl3d_sag_p4_iso_1.0

Routine		Prescan Normalize	Off
Nr. of slab groups	1	Normalize	Off
Slabs	1	B1 filter	Off
Position	Isocenter	Raw filter	Off
Orientation	Sagittal	Elliptical filter	Off
Phase enc. dir.	A >> P	Slice resolution	100%
Phase oversampling	0%	Slice partial Fourier	Off
Slice oversampling	0%	Geometry	
FoV read	256 mm	Nr. of slab groups	1
FoV phase	87.50%	Slabs	1
Slice thickness	4 mm	Position	Isocenter
TR	4.6 ms	Phase enc. dir.	A >> P
TE	2 ms	Phase oversampling	0%
Averages	1	Slice oversampling	0%
Concatenations	1	Slices per slab	44
Filter	None	System	
Coil elements	{head_neck}	Coil Combine Mode	Sum of Squares
Contrast		Auto Coil Select	Off
MTC	Off	Shim mode	Standard
Magn. preparation	None	Sequence	
Flip angle	6 deg	Introduction	On
Fat suppr.	None	Dimension	3D
Water suppr.	None	Elliptical scanning	On
SWI	Off	Phase stabilisation	Off
Averaging mode	Short term	Averaging mode	Short term
Measurements	1	Multi-slice mode	Interleaved
Reconstruction	Magnitude	Asymmetric echo	Off
Multiple series	Each measurement	Contrasts	1
Resolution		Bandwidth	480 Hz/Px
Base resolution	64	Flow comp.	No
Phase resolution	100%	Allowed delay	0 s
Phase partial Fourier	Off	RF pulse type	Fast
Interpolation	Off	Gradient mode	Normal
PAT mode	None	Excitation	Non-sel.
Image Filter	Off	RF spoiling	On
Distortion Corr.	Off		

Rf_sens_body
same as before, but coil elements = body

MPM protocol starting next page…

MPM_T1 TA:3:50 PAT:2 Voxel size:1.0×1.0×1.0 mm

Source protocol: Siemens\head\library_3D\t1_fl3d_sag_p4_iso_1.0

**Routine**	
Nr. of slab groups	1
Slabs	1
Position	Isocenter
Orientation	Sagittal
Phase enc. dir.	A >> P
Phase oversampling	0%
Slice oversampling	0%
FoV read	256 mm
FoV phase	87.50%
Slice thickness	1 mm
TR	18.0 ms
TE 1	2.46 ms
TE 2	4.92 ms
TE 3	7.38 ms
TE 4	9.84 ms
TE 5	12.30 ms
TE 6	14.76 ms
Averages	1
Concatenations	1
Filter	Distortion Corr.(3D)
Coil elements	HEA;HEP
**Contrast**	
MTC	Off
Magn. preparation	None
Flip angle	25 deg
Fat suppr.	None
Water suppr.	None
SWI	Off
Averaging mode	Short term
Measurements	1
Reconstruction	Magnitude
Multiple series	Each measurement
**Resolution**	
Base resolution	256
Phase resolution	91%
Phase partial Fourier	Off
Interpolation	Off
PAT mode	GRAPPA
Accel. factor PE	2
Ref. lines PE	24
Reference scan mode	Integrated
Image Filter	Off
Distortion Corr.	Off
Accel. factor 3D	1
Mode	3D
Unfiltered images	On
Prescan Normalize	On
Normalize	Off
B1 filter	Off
Raw filter	Off
Elliptical filter	Off
Slice resolution	91%
Slice partial Fourier	6/8
**Geometry**	
Nr. of slab groups	1
Slabs	1
Dist. factor	20%
Position	Isocenter
Phase enc. dir.	A >> P
Phase oversampling	0%
Slice oversampling	0%
Slices per slab	176
Multi-slice mode	Interleaved
Series	Interleaved
**System**	
Body	Off
Save uncombined	Off
Coil Combine Mode	Sum of Squares
**Sequence**	
Introduction	On
Dimension	3D
Elliptical scanning	On
Phase stabilisation	Off
Averaging mode	Short term
Multi-slice mode	Interleaved
Asymmetric echo	Off
Contrasts	6
Bandwidth 1	480 Hz/Px
Flow comp. 1	No
Readout mode	Bipolar
Allowed delay	0 s
RF pulse type	Fast
Gradient mode	Normal
Excitation	Non-sel.
RF spoiling	On

MPM_PD TA:3:50 PAT:2 Voxel size:1.0×1.0×1.0 mm
same as T1, but Flip angle = 4 deg

MPM_MT TA:7:52 PAT:2 Voxel size:1.0×1.0×1.0 mm
same as T1/PD, but TR = 37.0 ms, Flip angle = 6 deg, MTC = On (Contrast Card)

Philips protocol parameters starting next page…

B1 map

SmartSelect	yes
Coil 1 (exclude)	None
Uniformity	CLEAR
FOV FH (mm)	256
AP (mm)	240
RL (mm)	170
ACQ voxel size FH (mm)	4
AP (mm)	4
RL (mm)	10
Recon voxel size FH (mm)	1
AP (mm)	1
RL (mm)	5
Fold-over suppression	no
Slice oversampling	default
ENCASE enable	no
Reconstruction matrix	256
SENSE	no
CS-SENSE	no
Stacks	1
slices	34
slice orientation	sagittal
fold-over direction	AP
fat shift direction	F
Stack Offc. AP (P=+mm)	-24.0336266
RL (L=+mm)	2.33173585
FH (H=+mm)	17.2416954
Ang. AP (deg)	-1.09899402
RL (deg)	0.0476288982
FH (deg)	1.77370369
Free rotatable	no
Multi-chunk	no
Large table movement	no
PlanAlign	no
REST slabs	0
Shim Size AP (mm)	199.917542
RL (mm)	190
FH (mm)	225.881744
Offc. AP (P=+mm)	-24.7313118
RL (L=+mm)	2.24584699
FH (H=+mm)	22.8373032
Ang. AP (deg)	-1.09899402
RL (deg)	0.0476288982
FH (deg)	1.77370369
Patient position	head first
Patient body position	head first
Patient orientation	supine
Patient body orientation	supine
Scan type	Imaging
Scan mode	3D
technique	FFE
Contrast enhancement	T1
Acquisition mode	cartesian
Fast Imaging mode	none
Echoes	1
partial echo	no
shifted echo	no
TE	shortest
Flip angle (deg)	60

TR	user defined
(ms)	30
Halfscan	no
Water-fat shift	maximum
Shim	volume
ShimAlign	yes
mDIXON	no
Fat suppression	no
Water suppression	no
MTC	no
Research prepulse	no
Diffusion mode	no
Multi-transmit	no
SAR mode	high
B1 mode	default
SAR allow first level	yes
PNS mode	high
Gradient mode	default
SofTone mode	no
Cardiac synchronization	no
Respiratory compensation	no
Navigator respiratory comp	no
Flow compensation	no
fMRI echo stabilisation	no
NSA	1
Angio / Contrast enh.	no
Quantitative flow	no
Manual start	no
Dynamic study	no
Preparation phases	auto
Interactive F0	select
B1 field map	yes
field map technique	dual TR
TR extension	150
selective	yes
grad spoil factor	10
MIP/MPR	no
SWIp	no
Images	M, (3) no
Autoview image	M
Calculated images	B1, (3) no
Reference tissue	Grey matter
Recon compression	No
Preset window contrast	soft
Reconstruction mode	real time
Save raw data	no
Hardcopy protocol	no
Image filter	system default
Geometry correction	default
Elliptical k-space shutter	default
IF_info_seperator	1634755923
Research Options used	47
Total scan duration	03:36.1
Rel. SNR	312.167328
Act. TR/TE (ms)	30 / 2.2
ACQ matrix M x P	64 x 60
ACQ voxel MPS (mm)	4.00 / 4.00 / 10.0
REC voxel MPS (mm)	1.00 / 1.00 / 5.00

RF_sens_head

SmartSelect	yes
Coil 1 (exclude)	None
Uniformity	CLEAR
FOV FH (mm)	256
AP (mm)	240
RL (mm)	176
ACQ voxel size FH (mm)	4
AP (mm)	4
RL (mm)	4
Recon voxel size FH (mm)	1
AP (mm)	1
RL (mm)	4
Fold-over suppression	no
Slice oversampling	no
ENCASE enable	no
Reconstruction matrix	256
SENSE	no
CS-SENSE	no
Stacks	1
slices	44
slice orientation	sagittal
fold-over direction	AP
fat shift direction	F
Multi-chunk	no
Large table movement	no
PlanAlign	no
REST slabs	0
Patient position	head first
Patient body position	head first
Patient orientation	supine
Patient body orientation	supine
Scan type	Imaging
Scan mode	3D
technique	FFE
Contrast enhancement	T1
Acquisition mode	cartesian
Fast Imaging mode	none
Echoes	1
partial echo	no
shifted echo	no
TE	user defined
(ms)	2
Flip angle (deg)	6
TR	user defined
(ms)	4.6
Halfscan	yes
factor Y	0.625
factor Z	1
Water-fat shift	user defined
(pixels)	0.899999976
Shim	auto

mDIXON	no
Fat suppression	no
Water suppression	no
MTC	no
Research prepulse	no
Diffusion mode	no
Multi-transmit	no
SAR mode	high
B1 mode	default
SAR allow first level	yes
PNS mode	high
Gradient mode	maximum
SofTone mode	no
Cardiac synchronization	no
Respiratory compensation	no
Navigator respiratory comp	no
Flow compensation	no
fMRI echo stabilisation	no
NSA	1
Angio / Contrast enh.	no
Quantitative flow	no
Manual start	no
Dynamic study	no
Preparation phases	auto
Interactive F0	no
B1 field map	no
MIP/MPR	no
SWIp	no
Images	M, (3) no
Autoview image	M
Calculated images	(4) no
Reference tissue	Grey matter
Recon compression	No
Preset window contrast	soft
Reconstruction mode	immediate
Save raw data	no
Hardcopy protocol	no
Image filter	system default
Uniformity correction	no
Geometry correction	default
Elliptical k-space shutter	default
IF_info_seperator	0
Total scan duration	00:08.9
Rel. SNR	42.3680992
Act. TR/TE (ms)	4.6 / 2.0
ACQ matrix M x P	64 x 60
ACQ voxel MPS (mm)	4.00/4.00/4.00
REC voxel MPS (mm)	1.00/1.00/4.00
Scan percentage (%)	100
Act. slice gap (mm)	0
Act. WFS (pix) / BW (Hz)	0.898 / 483.4
Min. WFS (pix) / Max. BW (Hz)	0.082/5277.0
Min. TR/TE (ms)	4.0 / 1.54

RF_sens_body

same as RF_sens_head, except following settings:

SmartSelect	no
clinical modes	BODY_QUAD.Complete
Coil 1	Q-Body

MPM_T1

SmartSelect	yes
Coil 1 (exclude)	None
Uniformity	Synergy
FOV FH (mm)	256
AP (mm)	240
RL (mm)	176
ACQ voxel size FH (mm)	1
AP (mm)	1
RL (mm)	1
Recon voxel size FH (mm)	1
AP (mm)	1
RL (mm)	1
Fold-over suppression	no
Slice oversampling	default
ENCASE enable	no
Reconstruction matrix	256
SENSE	yes
P reduction (AP)	2
S reduction (RL)	1.25
CS-SENSE	no
Stacks	1
slices	176
slice orientation	sagittal
fold-over direction	AP
fat shift direction	F
Stack Offc. AP (P=+mm)	-30.3245182
RL (L=+mm)	4.56526184
FH (H=+mm)	16.5020771
Ang. AP (deg)	-1.09899402
RL (deg)	0.0476288982
FH (deg)	1.77370369
Free rotatable	no
Multi-chunk	no
Large table movement	no
PlanAlign	no
REST slabs	0
Patient position	head first
Patient body position	head first
Patient orientation	supine
Patient body orientation	supine
Scan type	Imaging
Scan mode	3D
technique	FFE
Contrast enhancement	T1
Acquisition mode	cartesian
Fast Imaging mode	none
Echoes	6
partial echo	no
shifted echo	no
TE first	user defined
(ms)	2.4000001
echospacing	user defined
(ms)	2.4000001
flyback	no
Flip angle (deg)	25
TR	user defined
(ms)	18
Halfscan	yes

factor Y	0.625
factor Z	1
Water-fat shift	user defined
(pixels)	0.899999976
Shim	auto
mDIXON	no
Fat suppression	no
Water suppression	no
MTC	no
Research prepulse	no
Diffusion mode	no
Multi-transmit	no
SAR mode	high
B1 mode	default
SAR allow first level	yes
PNS mode	high
Gradient mode	maximum
SofTone mode	no
Cardiac synchronization	no
Respiratory compensation	no
Navigator respiratory comp	no
Flow compensation	no
fMRI echo stabilisation	no
NSA	1
Angio / Contrast enh.	no
Quantitative flow	no
Manual start	no
Dynamic study	no
Preparation phases	auto
Interactive F0	no
B1 field map	no
MIP/MPR	no
SWIp	no
Images	M, (3) no
Autoview image	M
Calculated images	(4) no
Reference tissue	Grey matter
Recon compression	No
Preset window contrast	soft
Reconstruction mode	immediate
Save raw data	no
Hardcopy protocol	no
Image filter	system default
Uniformity correction	no
Geometry correction	default
Elliptical k-space shutter	default
IF_info_seperator	0
Total scan duration	03:37.4
Rel. SNR	2.27444863
Act. TR/TE1/delta TE (ms)	18 / 2.4 / 2.4
ACQ matrix M x P	256 x 240
ACQ voxel MPS (mm)	1.00/1.00/1.00
REC voxel MPS (mm)	1.00/1.00/1.00
Scan percentage (%)	100
Act. slice gap (mm)	5.96E-08
Act. WFS (pix) / BW (Hz)	0.907/478.7
Min. WFS (pix) / Max. BW (Hz)	0.329/1319.6
Min. TR/TE1/delta TE (ms)	17 / 2.0 / 2.4

MPM_PD and MPM_MT on next page...

MPM_PD

same as **MPM_T1**, except following settings:

Flip angle (deg)	4	Total scan duration	03:37.4

MPM_MT

same as **MPM_T1**, except following settings:

Flip angle (deg)	6	Research Options used	26
TR	user defined	Total scan duration	09:39.8
(ms)	48		
MTC	off res. multi		
nr repetitions	1		
angle	220		
duration	8		
frequency	1000		
